# Electron Push-Pull Effect of Benzotrithiophene-Based Covalent Organic Frameworks on the Photocatalytic Degradation of Pharmaceuticals and Personal Care Products

**DOI:** 10.3390/molecules30020336

**Published:** 2025-01-16

**Authors:** Hongguang Guo, Jiaqin He, Yixi Guo, Yunxi Chang, Haidong Ju, Yizhou Li

**Affiliations:** 1 School of Chemistry and Chemical Engineering, Kunming University, Kunming 650214, China; guohg0906@163.com (H.G.); changyx0925@163.com (Y.C.); 2 College of Physics Science and Technology, Kunming University, Kunming 650214, China; h1771658775@163.com (J.H.); guoyixi129@163.com (Y.G.); 3 Yunnan Engineering Technology Research Center for Plastic Films, Kunming University, Kunming 650214, China; 4 Yunnan Key Laboratory of Metal-Organic Molecular Materials and Device, Kunming University, Kunming 650214, China

**Keywords:** covalent organic frameworks, photocatalytic degradation, benzotrithiophene, pharmaceuticals and personal care products, triazine

## Abstract

A covalent organic framework (COF) has emerged as a promising photocatalyst for the removal of pharmaceutical and personal care product (PPCP) contaminants; however, high-performance COF photocatalysts are still scarce. In this study, three COF photocatalysts were successfully synthesized by the condensation of benzo[1,2-b:3,4-b′:5,6-b′′]trithiophene-2,5,8-tricarbaldehyde (BTT) with 4,4′,4′′-(1,3,5-triazine-2,4,6-triyl)trianiline (TAPT), 1,3,5-Tris(4-aminophenyl)benzene (TAPB), and 4,4′,4′′-nitrilotris(benzenamine) (TAPA), namely, BTT-TAPA, BTT-TAPB, and BTT-TAPT, respectively. The surface areas of BTT-TAPA, BTT-TAPB, and BTT-TAPT were found to be 800.46, 1203.60, and 1413.58 cm^2^∙g^−1^, respectively, providing abundant active sites for photocatalytic reactions. Under visible-light irradiation, BTT-TAPT exhibited the highest removal rate of tetracycline (TC), reaching 82.7% after 240 min. The superior photocatalytic performance of BTT-TAPT was attributed to its large specific surface area and the strong electron-acceptor properties of the triazine group. Electron paramagnetic resonance capture experiments and liquid chromatograph mass spectrometer analysis confirmed that superoxide radicals played a pivotal role in the degradation of TC and ciprofloxacin. Moreover, BTT-TAPT exhibited high stability and reproducibility during the photocatalytic degradation process. This study confirms that BTT-based COFs are a class of promising photocatalysts for the degradation of PPCPs in water, and their performance can be further optimized by tuning the structure and composition of the frameworks.

## 1. Introduction

Pharmaceuticals and personal care products (PPCPs) encompass various antibiotics, sanitizers, drugs, hormones, anti-inflammatory drugs, disinfectants, fragrances, soaps, etc. These compounds can disrupt the endocrine system, leading to imbalances in homeostasis or other adverse effects. In recent years, particularly during the COVID-19 pandemic, PPCPs have been increasingly detected in ground, surface, and drinking water. Consequently, PPCPs have potential risks to human health and ecological systems and are now regarded as a new category of water contaminants [1]. Specifically, antibiotics have caused significant damage to aquatic ecosystems, such as tetracycline (TC), ciprofloxacin (CIP) [2,3,4,5], and diuretic hydrochlorothiazide [6]. Therefore, developing an effective method to remove PPCPs from water is crucial [7].

At present, various methods have been developed for the removal of PPCPs from water, including ion exchange [8], membrane filtration [9], adsorption [10], reverse osmosis [11], bioengineering processes [12], metal-based catalysis [13], and photocatalysis [14]. Among them, photocatalysis stands out as a promising technique for the treatment of PPCPs in wastewater, owing to its environmental friendliness, high efficiency, safety, and absence of secondary pollution. As a metal-free photocatalyst, graphitic carbon nitride (g-C_3_N_4_) has been considered a strong competitor to the classic TiO_2_ photocatalyst due to its advantageous properties, including low cost and non-toxicity [15]. However, bulk g-C_3_N_4_ encounters several challenges, such as inadequate utilization of visible light, a restricted surface area, a rapid recombination rate of charge carriers, and low electrical conductivity, which collectively hinder its catalytic performance [16]. Therefore, an excellent photocatalyst should possess a high specific surface area and multiple active sites to enhance its activity.

Recently, covalent organic frameworks (COFs) have received significant attention due to their unique properties, such as high stability, pre-designed structures, tunable absorption bands, ordered porosity, and accessible functionality [17,18,19], which facilitate their broad applications in catalysis, gas separations, energy conversion, water harvesting, drug delivery, and antibacterial therapy [20,21,22,23,24,25]. As porous crystalline polymers, COFs typically feature π-π conjugated backbones with high charge carrier separation and a low band gap, which endows COFs with great potential for photocatalytic degradation. At first, researchers deposited inorganic semiconductors onto the surface of COF materials with a high specific surface area, enhancing their photocatalytic performance. Bi, JH introduced a novel hollow flower-ball-like nano heterojunction composed of a N-rich COF and BiOBr, which significantly enhanced TC-degradation efficiency, attributed to efficient photoinduced electron transfer and augmented separation of photogenerated charge carriers [26]. A novel COF heterojunction composite, TzDa/Ag/AgBr, can efficiently degrade TC and reduce Cr(VI) under visible light [27]. However, COFs with varying intensities of interfacial electric fields (IEFs) exhibit strong intrinsic charge separation and transfer capabilities, demonstrating excellent performance of photocatalytic degradation under light irradiation [28,29,30]. Yang YL et al. developed a triazine-functionalized fully conjugated COF with high chemical stability and superior carrier conduction, exhibiting high performance and reusability in the photocatalytic degradation of dyes [31]. The triazine-linked COFs could degrade 5-nitro-1,2,4-triazol-3-one, an insensitive explosive present in industrial wastewater. Currently, COFs are mainly used for photocatalytic hydrogen production, reduction in CO_2_, photocatalytic organic reaction transformations, photocatalytic environmental remediation, and photocatalytic degradation of organic dyes [32]. Nevertheless, there is a scarcity of research on COFs that are employed in the degradation of PPCPs, particularly antibiotics.

Benzotrithiophene (BTT), a stable aromatic ring compound, has been used as an electron-donated component for COFs due to its electron-rich triple thiophene moiety, n-π* transitions of long pairs on the sulfur atom, and favorable molecular orbital occupancies [33,34]. Moreover, its π-network facilitates exciton migration and charge carrier transport, which is crucial for preventing back-recombination of charges [35]. A two-dimensional (2D) imine-linked COF with BTT as an electron donor has been developed as a highly active blue-light-driven photocatalyst for selective aerobic sulfoxidation, highlighting the role of the electron push–pull effect in enhancing visible-light photocatalysis [36]. BTT-based COFs with spatially separated redox centers have achieved a breakthrough in hydrogen peroxide production from water and oxygen without sacrificial agents, demonstrating high selectivity, yield rates, and solar-to-chemical conversion efficiency [37]. Two COFs incorporating BTT as the electron donor had superior photocatalytic degradation efficiency for Rhodamine B [38]. Qin CC et al. optimized the photocatalytic performance of BTT-based COFs by adjusting the length of the aromatic chains, maintaining degradation rates for antibiotics above 85% [39]. Therefore, the separation and transfer of photogenerated excitons can be regulated by dipole moments.

**Figure 1:**
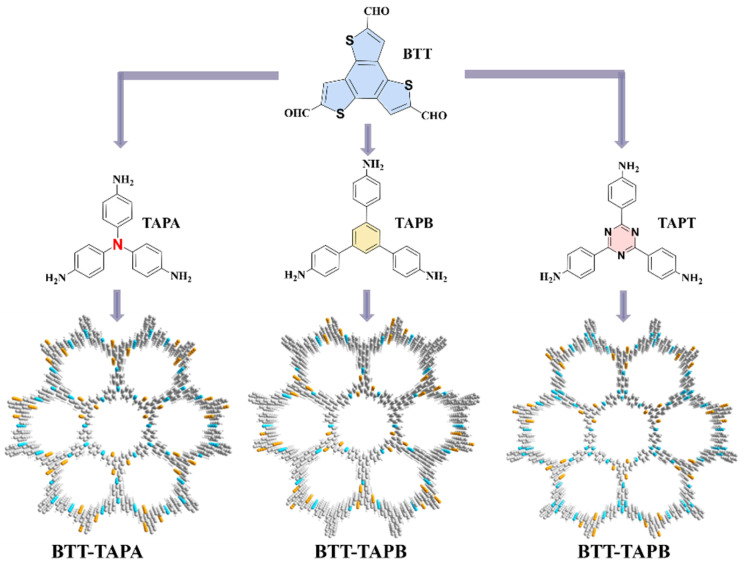
Synthesis of BTT-based COFs (BTT-TAPA, BTT-TAPB and BTT-TAPT).

In this study, we investigated the pivotal role of the IEF in BTT-based COFs through regulating the dipole moment. 4,4′,4″-(1,3,5-triazine-2,4,6-triyl)trianiline (TAPT), 1,3,5-Tris(4-aminophenyl)benzene (TAPB), and 4,4′,4″-nitrilotris(benzenamine) (TAPA) were utilized as functional groups of dipole moment regulation to modulate the electronic structure, pore size, and surface features of the framework. As illustrated in Figure 1 and Figures S1–S3, we successfully synthesized three BTT-based COFs (BTT-TAPA, BTT-TAPB, and BTT-TAPT) through the Schiff base reaction at 120 °C for 3 days. Their composition, chemical bonds, crystal structures, and morphology were meticulously analyzed by X-ray photoelectron spectroscopy (XPS), powder X-ray diffraction (PXRD), Fourier-transform infrared spectroscopy (FT-IR) and scanning electron microscopy (SEM), respectively. The visible-light-driven degradation reaction of TC and CIP was used to evaluate the photocatalytic activity of BTT-TAPA, BTT-TAPB, and BTT-TAPT. Among these samples, BTT-TAPT exhibited the most remarkable catalytic performance for TC and CIP degradation. Density functional theory (DFT) calculations confirmed that the IEF differences of the three COFs affected their photocatalytic degradation performance. The strategy of the dipole moment regulation for COFs offers a viable solution to design photocatalytic materials for mitigating water pollution.

## 2. Results

### 2.1. Structure and Morphology

**Figure 2:**
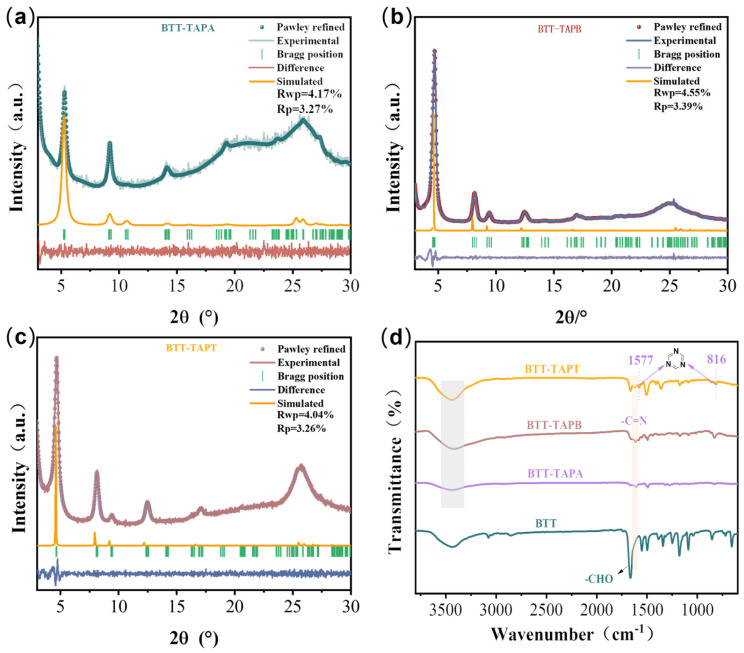
PXRD and Pawley refinement patterns of (**a**) BTT-TAPA, (**b**) BTT-TAPB, and (**c**) BTT-TAPT through Pawley refinement; (**d**) FT-IR spectra of BTT-TAPA, BTT-TAPB, and BTT-TAPT.

As shown in Figure 2a–c, the unit cell parameters and crystallinity of BBT-based COFs were confirmed by powder X-ray diffraction (PXRD) measurements with the assistance of computational simulation calculations. The PXRD patterns revealed the moderate crystallinity of BTT-TAPA and the high crystallinity of BTT-TAPB and BTT-TAPT. The strongest diffraction peaks of BTT-TAPA, BTT-TAPB, and BTT-TAPT can be assigned to the (100) lattice planes, occurring at 5.3, 4.7, and 4.7°, respectively. For BTT-TAPT, additional significant peaks were observed at 8.5, 9.4, and 12.4°, corresponding to (200), (210), and (220) facet reflections, respectively. Similarly, BTT-TAPB showed peaks at 8.1, 9.4, and 12.4°, which matched the (110), (200), and (210) lattice planes, respectively [36]. In contrast, BTT-TAPA displayed other distinct peaks at 9.2 and 14.1°, corresponding to the (200) and (210) lattice planes, respectively [37]. The broad peaks around 26° in all three BTT-based COFs were attributed to the (001) crystal plane, indicating the π-π stacking between COF layers [40,41]. Pawley refinement results showed that the simulated data based on the eclipse AA stacking model were well consistent with the experimental PXRD patterns, including peak position and relative intensity. As shown in Table 1, the unit cell parameters are determined to be a = b = 19.18 Å and c = 3.52 Å (R_wp_ = 4.175% and R_p_ = 3.27%) for BTT-TAPA, a = b = 21.35 Å and c = 3.50 Å (R_wp_ = 4.55% and R_p_ = 3.39%) for BTT-TAPB, and a = b = 22.20 Å and c = 3.49 Å (R_wp_ = 4.04% and R_p_ = 3.26%) for BTT-TAPB. Therefore, it can be inferred that three imine-linked 2D COFs based on BTT have been successfully synthesized.

The compositions of BTT-based COFs were checked by FTIR, as shown in Figure 2d and Figure S4. Compared with the BTT raw material, the FTIR spectra of BTT-TAPA, BTT-TAPB, and BTT-TAPT COFs exhibit a significant reduction in C=O stretching vibrations at 1666 cm^−1^. As illustrated in Figure S4, the NH_2_ stretching vibrations within the range of 3325 to 3460 cm^−1^ are evident in the raw material (TAPA, TAPB, and TAPT) FTIR spectra; however, they can hardly be detected in the BTT-based COF FTIR spectra. In addition, the characteristic C=N stretching vibrations at approximately 1624 cm^−1^ appear in the BTT-based COF FTIR spectra. Therefore, it can be inferred that BTT-TAPA, BTT-TAPB, and BTT-TAPT COFs had been successfully synthesized by an amine–aldehyde condensation reaction [42].

**Figure 3:**
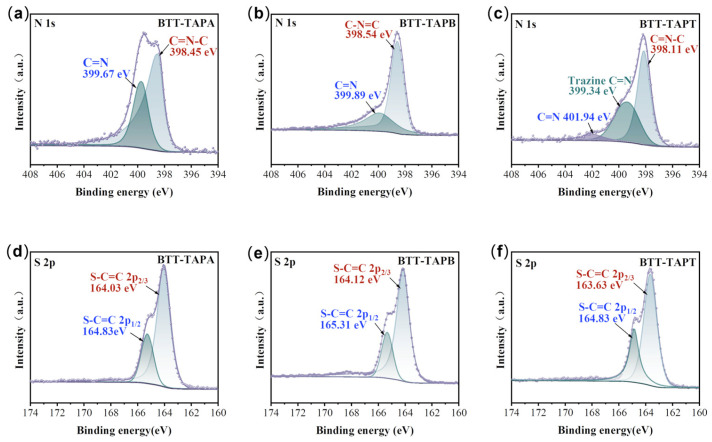
XPS spectra: (**a**–**c**) N 1s and (**d**–**f**) S 2p for BTT-TAPA, BTT-TAPB, and BTT-TAPT.

To further confirm the compositions and structure, X-ray photoelectron spectroscopy (XPS) was utilized for the characterization of BTT-based COFs. As shown in Figure 3, carbon, nitrogen, and sulfur atoms have been confirmed to exist in BTT-TAPA, BTT-TAPB, and BTT-TAPT COFs. In the high-resolution region of N 1s spectra, all BTT-based COFs exhibited two peaks at 399.34~399.89 eV and 398.11~398.54 eV (Figure 3a–c), which were ascribed to C-N and C=N bonds in the C=N-C groups, respectively [37,43,44]. Moreover, another peak at 401.94 eV appears in the BTT-TAPT XPS spectrum, which is due to the satellite peaks [44,45,46]. As shown in Figure 3d–f, all S 2p XPS bands of BTT-COFs could be deconvoluted into two peaks at about 163.03~164.12 eV and 164.83~164.12 eV due to S 2p_3/2_ and S 2p_1/2_, respectively. The XPS results are consistent with that of FTIR and further confirm the successful formation of imine-linked BTT-based COFs [47].

**Figure 4:**
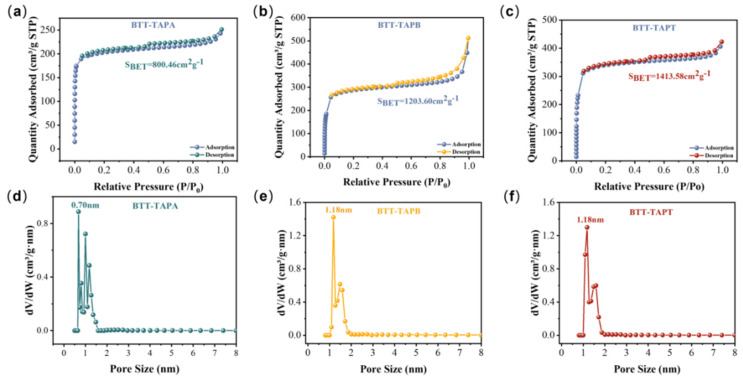
N_2_ adsorption/desorption isotherms of (**a**) BTT-TAPA, (**b**) BTT-TAPB, and (**c**) BTT-TAPT. Pore distribution curves of (**d**) BTT-TAPA, (**e**) BTT-TAPB, and (**f**) BTT-TAPT.

The porous structure of BTT COFs was characterized by their specific surface areas and pore sizes, which were verified by N_2_ adsorption–desorption measurements after activation. As shown in Figure 4a–c, the Brunauer–Emmett–Teller (BET) surface areas of BTT-TAPA, BTT-TAPB, and BTT-TAPT were calculated to be 800.46, 1203.60, and 1413.58 cm^2^∙g^−1^, respectively. According to the pore model, the pore size distribution profiles indicated that the pore sizes of BTT-TAPA, BTT-TAPB, and BTT-TAPT were 0.70, 1.18, and 1.18 nm (Figure 4e,f), respectively. Therefore, the high specific surface area and nanoscale pore structure provide more active sites, facilitating the rapid migration of photogenerated charge carriers to the surface, where they can participate in photocatalytic oxidation-reduction reactions.

**Figure 5:**
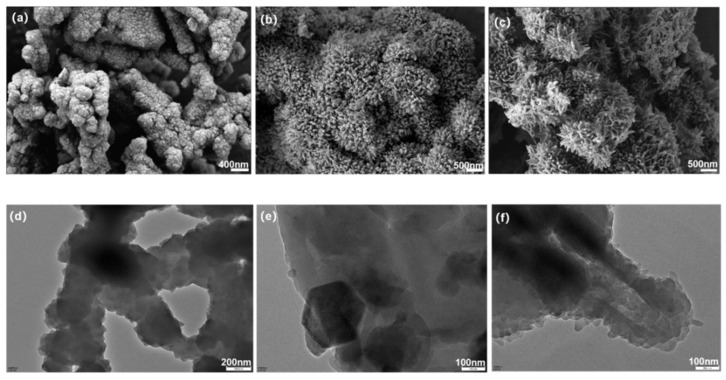
SEM images of (**a**) BTT-TAPA, (**b**) BTT-TAPB, and (**c**) BTT-TAPT. TEM images of (**d**) BTT-TAPA, (**e**) BTT-TAPB, and (**f**) BTT-TAPT.

As shown in Figure 5, the morphologies and microstructure of BTT-based COFs were characterized by scanning electron microscopy (SEM) and transmission electron microscopy (TEM). Figure 5a shows that BTT-TAPA is composed of ginger-shaped aggregates, with small polyhedral particles attached to the surface. As depicted in Figure 5b,c, both BTT-TAPB and BTT-TAPT have a sea-urchin-like morphology. The petal-like rods of BTT-TAPT are sparser and longer than that of BTT-TAPB. The TEM images of BTT-TAPB and BTT-TAPT show that some packaging materials exist inside these materials. Especially, a regular hexagonal sheet-like particle is shown in Figure 5e, which can be attributed to the crystalline morphology of BTT-TAPB with a hexagonal system [48,49,50]. Figure 5f exhibits that BTT-TAPT has a large hollow tube, which means that BTT-TAPT has a larger specific surface area.

### 2.2. Optoelectronic Properties

**Figure 6:**
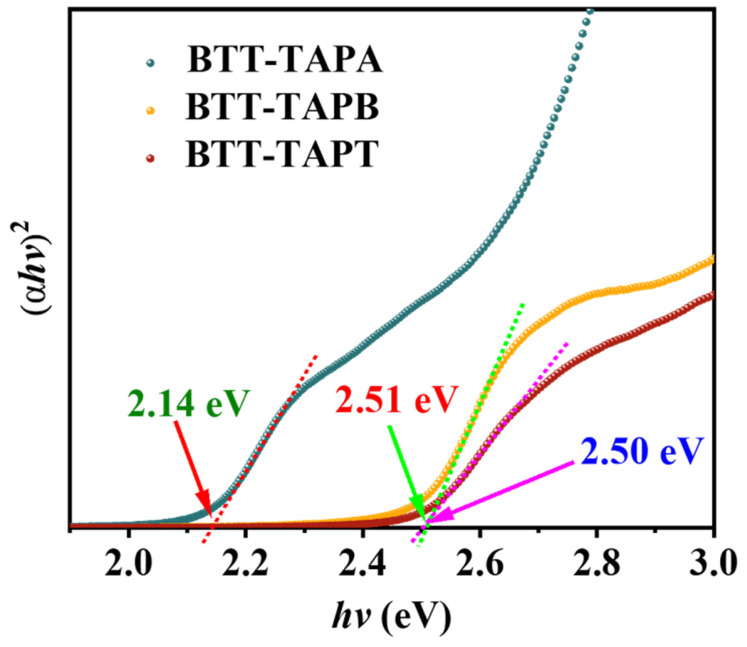
Tauc plots of BTT-TAPA, BTT-TAPB, and BTT-TAPT.

Photocatalytic degradation performance is closely related to light-harvesting and redox capacity, which can be preferentially evaluated by ultra-violet/visible (UV–Vis) absorption diffuse reflectance spectroscopy (DRS). The optical band gap is calculated using the following equation [51,52,53]:

(1) $${\left(\alpha h\nu \right)}^{n/2}=A(hv-Eg)$$

where *α* denotes the absorbance index, *h* is Planck’s constant, γ represents the frequency of light, *A* is the Coulomb constant, and *E_g_* denotes the gap energy of the absorption band. The value of *n* depends on the type of semiconductor, where *n* = 1 and 4 represent direct and indirect transitions, respectively. By linear fitting graphs, it can be easily determined that *n* = 1. As shown in Figure 6**,** the band gaps of BTT-TAPA, BTT-TAPB, and BTT-TAPT were calculated to be 2.14, 2.51, and 2.50 eV, respectively, which are far less than those of classical photocatalysts TiO_2_ (3.2 eV) and ZnO (3.1 eV) [54]. Therefore, these BTT-based COFs are excellent candidates for visible-light-promoted catalysts [55].

**Figure 7:**
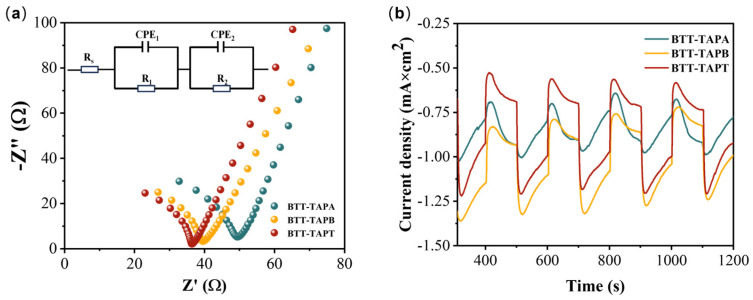
(**a**) EIS Nyquist plots of BTT-TAPA, BTT-TAPB, and BTT-TAPT with the simulated circuit diagrams shown in the inset. (**b**) Periodic on/off photocurrent responses of BTT-TAPA, BTT-TAPB, and BTT-TAPT under visible-light irradiation.

Electrochemical impedance spectroscopy (EIS) and transient photo-current responses (TPCRs) were conducted to further reveal the charge migration behavior. A small semicircular radium in the EIS curve at a high frequency usually indicates a low charge transfer resistance (RCT), suggesting rapid electron transfer at the material interface [37,56]. In Figure 7a, the EIS Nyquist plot of BTT-TAPT exhibited the lowest charge transfer resistance, facilitating the separation and migration of photogenerated electrons and holes [57]. As shown in Figure 7b, BTT-TAPA, BTT-TAPB, and BTT-TAPT all displayed a photocurrent response under visible-light irradiation from a xenon lamp, confirming their excellent photocatalytic performance. Specially, BTT-TAPT exhibited the strongest photocurrent corresponding signal among them, which suggested that it had excellent performance in the photocatalytic degradation of PPCPs.

**Figure 8:**
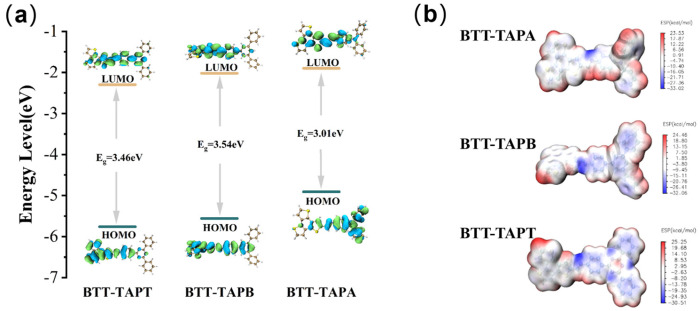
(**a**) Calculated orbital distributions of BTT-TAPT, BTT-TAPB, and BTT-TAPA. (**b**) ESP maps of BTT-TAPA, BTT-TAPB, and BTT-TAPT.

The photocatalytic reaction is a multi-step chemical reaction involving several intermediates. To better understand the reaction mechanism, it is essential to examine the frontier orbital distributions of the highest occupied molecular orbital (HOMO) and lowest unoccupied molecular orbital (LUMO) for the three BTT-based COFs, which were obtained by density functional theory (DFT) calculations at the Becke-3–Lee–Yang–Parr (B3LYP)/6-31G level. As shown in Figure 8, the HOMO of BTT-TAPA is primarily localized in the electron-donor triphenylamine unit, while the LUMO is spread across the electron-acceptor BTT part, indicating an obvious electron transfer from triphenylamine to the BTT group. For BTT-TAPB, the HOMO and LUMO are predominantly concentrated on the BTT unit, with only a slight electron transfer from TAPB to BTT during the electron transition from HOMO to LUMO. Although the frontier orbital distributions of the HOMO and LUMO in BTT-TAPT are similar to those of BTT-TAPB, electron migration in BTT-TAPT occurs in the opposite direction, from BTT to TAPT [47,58,59]. The HOMO energy levels were calculated to be −4.91, −5.56, and −5.76 eV for BTT-TAPA, BTT-TAPB, and BTT-TAPT, respectively. Therefore, it can be inferred that BTT-TAPT, with the lowest HOMO energy level, had strong oxidation potential. The LUMO energy levels were −1.90, −2.02, and −2.30 eV for BTT-TAPA, BTT-TAPB, and BTT-TAPT, respectively, yielding corresponding energy gaps of 3.01, 3.54, and 3.46 eV. The electrostatic potential (ESP) distribution shows that the electron-rich region is concentrated on the nitrogen atoms in the C-N=C band and TPAT groups (Figure 8b), indicating that BTT-TAPT offers more activation sites for oxidation reactions [60,61,62,63,64,65].

### 2.3. Photocatalytic Performance

**Figure 9:**
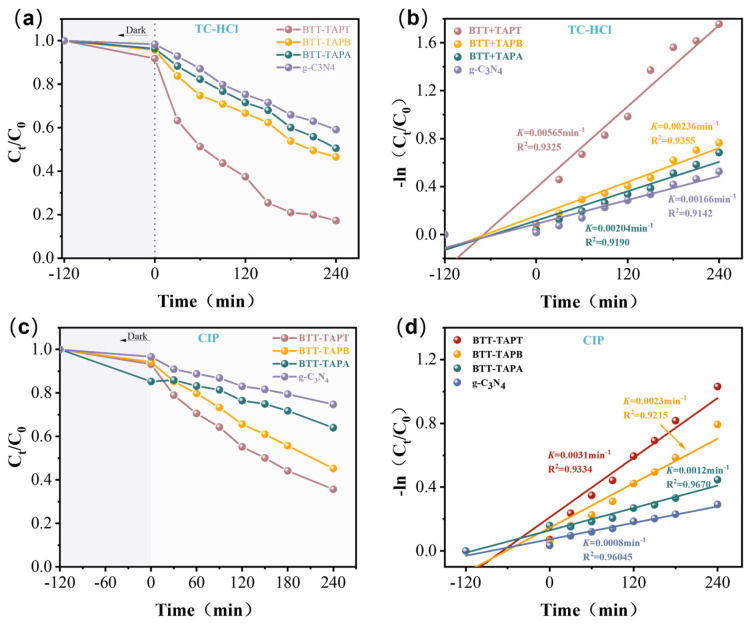
(**a**) Removal curves of TC (50 mg/L). (**b**) Pseudo-first-order reaction kinetics for the degradation of TC (50 mg/L) by BTT-TAPT (10 mg). (**c**) Removal curves of CIP (50 mg/L). (**d**) Pseudo-first-order reaction kinetics for the degradation of CIP (50 mg/L) by BTT-TAPT (10 mg).

To evaluate the photocatalytic performance of the BTT-based COFs, the degradation of various PPCPs, including TC and CIP antibiotics, was conducted under visible-light irradiation. TC demonstrated high photostability under visible light, with negligible degradation in the absence of a photocatalyst [66]. As shown in Figure 9a, after 240 min of visible light, the removal rate of TC was calculated to be 40.9, 49.5, 53.5, and 82.7% for g-C_3_N_4_, BTT-TAPA, BTT-TAPB, and BTT-TAPT, respectively. Compared with g-C_3_N_4_, BTT-TAPT with a porous structure showed a higher removal rate for TC, owing to its larger specific surface area [67]. Moreover, BTT-TAPT displayed a higher removal rate than BTT-TAPA and BTT-TAPB, which is attributed to the strong electron-acceptor properties of the TAPT group. The effects of the surface area and TAPT group on the photocatalytic degradation of TC were further investigated by the pseudo-first-order reaction kinetics model. As shown in Figure 9b, the degradation-rate constant (*k*) of g-C_3_N_4_, BTT-TAPA, BTT-TAPB, and BTT-TAPT are 0.0166, 0.0204, 0.0236, and 0.0565 min^−1^, respectively. The *k* value of BTT-TAPT was about 3.4-times higher than that of g-C_3_N_4_, and significantly greater than that of BTT-TAPA and BTT-TAPB. Moreover, the degradation effect of BTT-TAPT on TC is significantly higher than that of the other compounds listed in Table 2 [68,69,70,71,72,73,74,75,76,77]. The enhanced photocatalytic performance of BTT-TAPT can be attributed to its large surface area and electron-acceptor group, which promote photoinduced charge separation and oxidation. These results indicate that BTT-TAPT exhibits excellent photocatalytic performance. To assess the stability of the BTT-TAPT photocatalyst, cyclic catalysis experiments were carried out. After the catalytic degradation of TC, the BTT-TAPT photocatalyst was collected by centrifugation, washed sequentially with ionized water, ethanol, and acetone, and then dried in a vacuum oven at 80 °C for 12 h. The dried photocatalytic material was reused in subsequent TC-degradation experiments. The BTT-TAPT photocatalyst was successfully reused four times for photocatalytic degradation of TC, whose removal rates were 79.5, 77.8, 76.0, and 75.2% in the order Figure S6. The slight decrease in degradation efficiency may be due to the loss of some BTT-TAPT photocatalysts or incomplete washing during the washing process, resulting in partial pore clogging. To further verify the photocatalytic degradation ability of BTT-based COFs on PPCPs, the photocatalytic degradation of CIP was performed under the same experimental conditions as TC. Figure 9c shows the removal rates of CIP were 35.7, 45.3 63.4, and 74.7% for g-C_3_N_4_, BTT-TAPA, BTT-TAPB, and BTT-TAPT, respectively. The degradation-rate constant (*k*) for these samples was 0.00166, 0.00204, 0.00236, and 0.00565 min^−1^, respectively. The photocatalytic performance of BTT-TAPT still far exceeds that of g-C_3_N_4_, BTT-TAPA, and BTT-TAPB. Nevertheless, the high removal rate and good recycling characteristics suggest that BTT-TAPT is a promising photocatalyst for the removal of PPCPs.

### 2.4. Photocatalytic Degradation Mechanism

**Figure 10:**
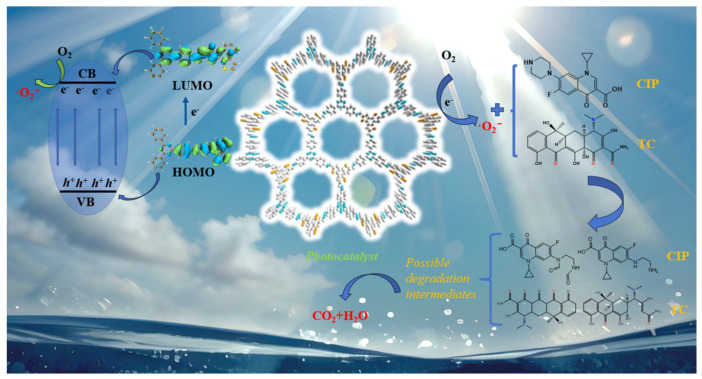
Photocatalytic degradation mechanism diagram.

The photocatalytic degradation mechanism of TC is shown in Figure 10. The photocatalytic degradation process was investigated through active species capture experiments and electron paramagnetic resonance (EPR) spectroscopy. 5,5-dimethyl-1-pyrroline-N-oxide (DMPO) was used as the spin-trapping agent to detect the superoxide radicals (•O_2_^−^) during the photocatalytic degradation process. Without light, the EPR spectrum did not show any discernible signal peaks (Figure S7). After exposure to a 400 W xenon lamp for 5 min, the EPR spectrum of BTT-TAPT displayed six distinct peaks in the range of 3480 to 3540 G, which correspond to the characteristic signal of •O_2_^−^ [78]. Since •O_2_^−^ is a key intermediate in the photocatalytic degradation of TC, the EPR results confirmed that the BTT-TAPT COF could produce •O_2_^−^ and degrade TC under visible light [79,80]. To further elucidate the degradation mechanism of TC by BTT-TAPT catalysts, the liquid chromatograph mass spectrometer (LC-MS) technique was used to examine the superoxide radical complex [81,82]. As shown in Figure S8, the LC-MS analysis revealed a prominent peak at 185.0475, which closely matched the theoretical value (185.0449) for the superoxide radical complex (C_16_H_12_KNO_3_^•+^). Therefore, under sunlight, BTT-TAPT COFs can combine with O_2_ to form •O_2_^−^ radicals, which oxidize and degrade TC.

## 3. Discussion

In conclusion, three BTT-based 2D COFs, namely, BTT-TAPA, BTT-TAPB, and BTT-TAPT, were successfully constructed for understanding the photochemical degradation of PPCPs by modulating the electron distribution and transfer directionality. The BET surface areas were found to be 800.46, 1203.60, and 1413.58 cm^2^∙g^−1^ for BTT-TAPA, BTT-TAPB, and BTT-TAPT, respectively, providing abundant active sites for photocatalytic oxidation-reduction reactions. EIS and TPCR measurements revealed that the BTT-TAPT COF had the lowest charge transfer resistance and the strongest photocurrent response. DFT calculations demonstrated that functional groups (TAPA, TAPB, and TAPT) bound to BTT could effectively regulate electron distribution and transfer. Owing to its large specific surface area and the strong electron-acceptor TAPT, BTT-TAPT exhibited the highest removal rate for TC, reaching 82.7% degradation after 240 min of visible-light exposure. To verify the degradation mechanism, LC-MS analysis identified a prominent peak at 185.0475, indicating the presence of a superoxide radical complex. EPR capture experiments further confirmed that superoxide radicals played a pivotal role in mediating the degradation of TC and CIP. The stability and reproducibility of BTT-TAPT as a photocatalyst was demonstrated through cyclic catalysis tests. This study confirms that BTT-based COFs are promising photocatalysts, with their photocatalytic performance modulated by functional groups, offering a potential approach for designing high-efficiency photocatalysts to degrade PPCPs.

## 4. Materials and Methods

Three 2D covalent organic frameworks were obtained by the aldimine condensation between BTT and TAPA, TAPB, and TAPT, respectively. Detailed information on the chemical reagents, instruments, and methods used for polymer characterization is provided in the Supplementary Materials.

### 4.1. Synthesis of BTT-TAPT

BTT (0.05 mmol) and TAPT (0.05 mmol) were weighed and placed into 10 mL Schlenk tubes. Subsequently, mesitylene (0.7 mL) and 1,4-dioxane (0.3 mL) were pipetted into the Schlenk tubes, and the mixture was sonicated for 15 min at room temperature. Acetic acid aqueous solution (0.1 mL, 6 M) was then added to the Schlenk tubes, and the mixture was sonicated again for 30 min. The tubes were degassed by three freeze–pump–thaw cycles and then sealed. After heating at 120 °C for 3 days, precipitates appeared in the tubes. The precipitates were filtered and washed sequentially with dimethylformamide, tetrahydrofuran, and acetone. Finally, the BTT-BAPT sample was dried in a vacuum oven at 80 °C for 12 h.

### 4.2. Synthesis of BTT-TAPB

BTT (0.05 mmol) and TAPB (0.05 mmol) were weighed and placed into 10 mL Schlenk tubes. Subsequently, mesitylene (0.7 mL) and 1,4-dioxane (0.3 mL) were added to the Schlenk tubes, and the mixture was sonicated for 30 min at room temperature. Then, an acetic acid aqueous solution (0.1 mL, 6 M) was introduced, and the mixture was sonicated again for 15 min. The tubes were degassed by three freeze–pump–thaw cycles and then sealed. The reaction was carried out at 120 °C for 3 days, during which precipitates formed. The precipitates were filtered, and then washed sequentially with dimethylformamide, tetrahydrofuran, and acetone. Finally, the BTT-BAPB sample was dried in a vacuum oven at 80 °C for 12 h.

### 4.3. Synthesis of BTT-TAPA

BTT (0.05 mmol) and TAPA (0.05 mmol) were weighed and placed into 10 mL Schlenk tubes. Subsequently, 1,2-dichlorobenzene (0.7 mL) and n-butanol (0.3 mL) were pipetted into the Schlenk tubes, and the mixture was sonicated for 15 min at room temperature. Afterward, an acetic acid aqueous solution (0.1 mL, 6 M) was added, and the mixture was sonicated again for 15 min. The tubes were degassed by three freeze–pump–thaw cycles and then sealed. After heating at 120 °C for 3 days, precipitates appeared in the tubes. The precipitates were filtered and then washed sequentially with dimethylformamide, tetrahydrofuran, and acetone. Finally, the BTT-BAPA sample was dried in a vacuum oven at 80 °C for 12 h.

### 4.4. Photocatalytic Degradation of TC

BTT-TAPA (10 mg), BTT-TAPB (10 mg), BTT-TAPT (10 mg), and g-C_3_N_4_ (10 mg) were individually weighed and placed into four quartz tubes, each containing 50 mL of aqueous solution with 50 ppm TC. These solutions were magnetically stirred in the dark for 2 h to allow TC to reach adsorption–desorption equilibrium. Photocatalytic degradation of TC was initiated under irradiation from a 300 W xenon lamp (λ > 420 nm). Samples were taken every 30 min and filtered through a 0.22 µm membrane. The TC concentration (C_t_) in the filtrate was measured using a UV-Vis spectrophotometer. Degradation efficiency was evaluated by the ratio of C_t_ to C_0_ (TC initial concentration). The degradation rate (k, min^−1^) of the TC system was calculated using the pseudo-first-order kinetic model, ln(C_t_/C_0_) = −kt.

### 4.5. Photocatalytic Degradation of CIP

BTT-TAPA (10 mg), BTT-TAPB (10 mg), BTT-TAPT (10 mg), and g-C_3_N_4_ (10 mg) were individually weighed and added to four quartz tubes, each containing 50 mL of aqueous solution with 50 ppm CIP. These solutions were magnetically stirred in the dark for 2 h to allow CIP to reach adsorption–desorption equilibrium. The photocatalytic degradation reaction of CIP was carried out under irradiation from a 300 W xenon lamp (λ > 420 nm). Samples were collected every 30 min and filtered through a 0.22 µm membrane. The CIP concentration (C_t_) in the filtrate was measured by a UV-Vis spectrophotometer. Degradation efficiency was evaluated by the ratio of C_t_ to C_0_ (CIP initial concentration). The degradation rate (k, min^−1^) of the CIP system was calculated using the pseudo-first-order kinetic model, ln(C_t_/C_0_) = −kt.

## Figures and Tables

**Table 1:** Lattice parameters for BTT-TAPA, BTT-TAPB, and BTT-TAPT.

Model	a (Å)	b (Å)	c (Å)	α	β	γ	R_wp_	R_P_
BTT-TAPA	19.18	19.18	3.52	90°	90°	120°	4.17%	3.27%
BTT-TAPB	21.35	21.35	3.50	90°	90°	120°	4.55%	3.39%
BTT-TAPT	22.20	22.20	3.49	90°	90°	120°	4.04%	3.26%

**Table 2:** Photocatalysts for TC degradation.

Sample	Dosage (g/L)	Concentration (mg/L)	Time (min)	Removal Rate (%)	Year	Ref.
Bi/BiVO_4_	0.5	10	60	74.7%	2019	[68]
Ag_3_PO_4_/5GO	0.25	10	30	69.8%	2019	[69]
CdS	0.4	100	60	80.0%	2020	[70]
PS/MNPs	0.05	50	240	74.4%	2020	[71]
mpg-C_3_N_4_–ZIF-8	0.5	40	180	74.8%	2020	[72]
ZIS-TCN/3	0.1	20	60	86.1%	2021	[73]
g-C_3_N_4_/Ag/AgBr-8%	0.32	20	30	57.5%	2022	[74]
US/BiVO_4_/PMS	>1	20	60	78.6%	2023	[75]
W1-TiO_2_-Et	0.4	50	360	77.2%	2024	[76]
BiOBr/Bi_2_O_2_CO_3_	0.2	40	40	63.0%	2024	[77]
BTT-TAPT	0.1	50	240	82.7%	2024	this work

## Data Availability

The experimental data used to support the results of this study are available in the article and in the Supplementary Materials.

## Supplementary Materials

The following supporting information can be downloaded at: https://www.mdpi.com/article/10.3390/molecules30020336/s1, Figures S1–S3: Sample synthesis flowchart, Figure S4: FT-IR spectra of samples, Figure S5: XPS spectra of BTT-TAPA, BTT-TAPB, and BTT-TAPT, Figure S6: Repetitive photodegradation of BTT-TAPT on TC, Figure S7: EPR spectra of BTT-TAPT, Figure S8: High-resolution mass spectrometry of DMPO with superoxide radicals.
