# Supplementary material for: Electron Push-Pull Effect of Benzotrithiophene-Based Covalent Organic Frameworks on the Photocatalytic Degradation of Pharmaceuticals and Personal Care Products
Source: Molecules. 2025 Jan 16;30(2):336. doi: 10.3390/molecules30020336 (PMC11767667; doi:10.3390/molecules30020336)
Supplement: Supplementary file 1 [file molecules-30-00336-s001.zip › molecules-3423861-supplementary.pdf]

Supporting Information

Electron Push–Pull Effect of Benzotrithiophene−Based Covalent Organic Frameworks on the Photocatalytic Degradation of Pharmaceuticals and Personal Care Products.

1.Materials

1.1 Reagents and solvents

Benzo[1,2-b:3,4-b':5,6'']trithiophene-2,5,8-tricarbaldehyde (BTT), ciprofloxacin (CIP), tetracycline (TC), and 4,4',4''-(1,3,5-Triazine-2,4,6-triyl)trianiline (TAPT) reagents were obtained from Shanghai Macklin Biochemical Technology Co., Ltd. 4,4',4''-nitrilotris(benzenamine) (TAPA) and 1,3,5-tri(4-aminophenyl)benzene (TAPB) reagents were purchased from Shanghai Haohong Scientific Co., Ltd. Organic solvents, including 1,2-dichlorobenzene (o-DCB), n-butanol (n-BuOH), ethanol, acetone, tetrahydrofuran (THF), 1,3,5-Trimethylbenzene, N-dimethylformamide (DMF), 1,4-dioxane, and acetonitrile, were obtained from Aldric Chemicals. Acetic acid and melamine were purchased from the Tianjin Damao chemicals reagent factory. All reagents and solvents were used without further purification.

1.2 Synthesis of g-C_3N_4

The melamine was put into a tube furnace, and sintered at 550 °C for 4 h. After cooling to room temperature, the sample was washed sequentially with hot water and ethanol, and then dried in a vacuum oven at 80 °C for 10 h. The g-C_3N_4 sample was obtained.

2. Instrumentations

Powder X-ray diffraction (PXRD) data were collected by a Bruker D8 advance powder diffractometer with Ni filtered Cu Kα radiation at a scan rate of 2°/min. Fourier transform infrared (FT-IR) spectra were recorded with a Thermo Fisher Scientific Nicolet iN10 between 400-4000 cm^{-1}. Scanning electron microscopy (SEM) measurements were performed on a ZEISS Sigma 300 field emission scanning electron microscope. Transmission electron microscope (TEM) characterizations were

conducted using JEOL JEM-F20. The ultraviolet-visible diffusion reflectance spectra (UV-vis-DRS) were recorded by a Shimadzu UV-3600i Plus in the wavelength range of 200-800 nm at room temperature. The Shimadzu UV-2450 spectrophotometer was used for UV-visible diffuse reflectance measurements to assess antibiotic degradation. The specific surface areas and pore-size distributions were determined by N_2 physisorption using a Micromeritics ASAP 2460 automated system, applying the Brunauer-Emmet-Teller (BET) method. BTT-TAPA was degassed under vacuum ($<1\times10^{-5}$ bar) in the Micromertics system at 100 °C for 12 h prior to N_2 physisorption. BTT-TAPB and BTT-TAPT were degassed under vacuum ($<1\times10^{-5}$ bar) in the Micromertics system at 200 ℃ for 3 h prior to N_2 physisorption. Electrochemical impedance spectroscopy (EIS) data and response photocurrent tests were performed on the materials using the Autolab PGSTAT302N electrochemical workstation (Metrohm Autolab). Electron paramagnetic response (EPR) spectra was obtained using EPR200-Plus at room temperature. High resolution mass spectrometry (HRMS) were determined on a Thermo ScientificTM Orbitrap ExplorisTM 120 Mass Spectrometer.

3. Electrochemical measurements

Electrochemical measurements were carried out on the Autolab PGSTAT302N electrochemical workstation with the BTT-based COFs as the working electrode, an Ag/AgCl (saturated AgCl) as the reference electrode, and a platinum wire as the counter electrode. The electrolyte was a Na_2SO_4 aqueous solution (0.2 M). Photocatalyst (3 mg) was first dispersed in 1 mL 0.2% Nafion, and then the samples were coated on a ITO glass substrate. The sample was dried under an infrared lamp for 5 minutes prior to electrochemical impedance spectroscopy (EIS) and photocurrent response tests. For the photocurrent response test, a 300 W xenon lamp was used, with the sample positioned 20 cm away from the lamp. The voltage was stabilized for 200 s before the material was alternately illuminated and darkened in 100 s intervals.

4. Electron paramagnetic resonance (EPR) measurements

Spin trapping EPR tests were conducted using a domestically produced EPR200-Plus instrument at room temperature. 5,5-Dimethyl-1-pyrroline N-oxide (DMPO) was employed as a spin trapping agent to detect $\cdot O_2^-$. The catalysts (3 mg) were dispersed into a MeOH/water mixture (9/1 *v/v*, 2 mL) containing DMPO (0.4 mmol). One milliliter of the solution was added to a quartz photocatalytic reactor tube. A 400 W Xe lamp was used as the light source, and the signals were recorded for 0 and 5 minutes under illumination.

5. High-resolution mass spectra (HRMS) measurements

A 50 ppm TC solution was prepared in a 5 ml volume. The catalyst, at a concentration of 2 mg/ml, should be added to this solution, which should be illuminated for 10 minutes using a 400 W Xe lamp. Following illumination, the solution should be filtered through a 0.22 μm organic filtration membrane. A 22-micron organic filtration membrane should be prepared and available for use. The specifications for 1.5 mL glass vials should be obtained, and 1 mL of chromatographically pure acetonitrile should be added to the vial. Subsequently, 1 to 2 drops of the standby solution should be introduced into the vial. The vial should then be capped and allowed to stand for a specified period to enable the solution to interact with the sample.

6. Software calculation

The HOMO and LUMO for BTT-based COFs were calculated using Gaussian 16 with (B3LYP)/6-31G level .

7. Synthesis of COFs

7.1 Synthesis of BTT-TAPA

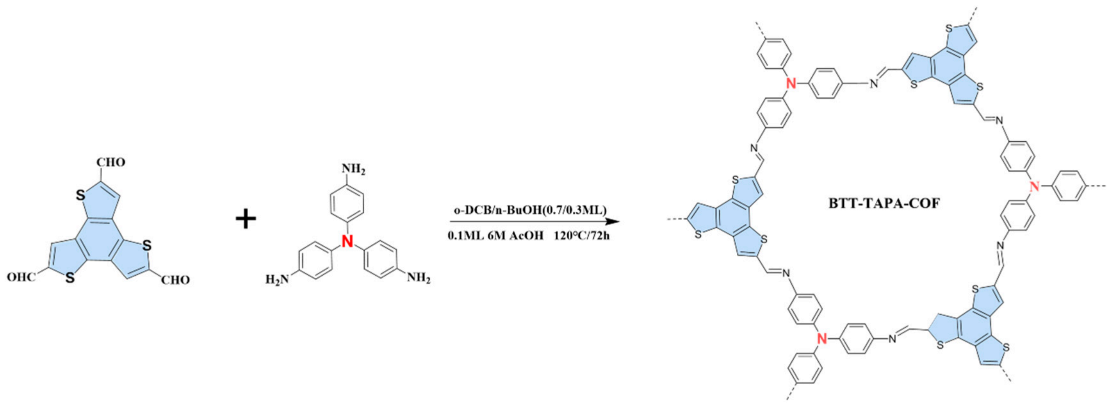

Figure S1. Synthesis of BTT-TAPA

7.2 Synthesis of BTT-TAPB

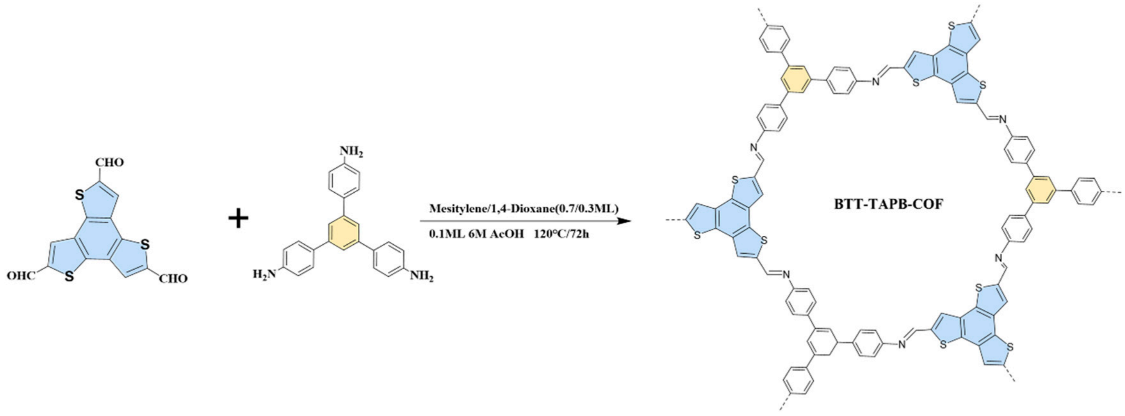

Figure S2. Synthesis of BTT-TAPA

7.3 Synthesis of BTT-TAPT

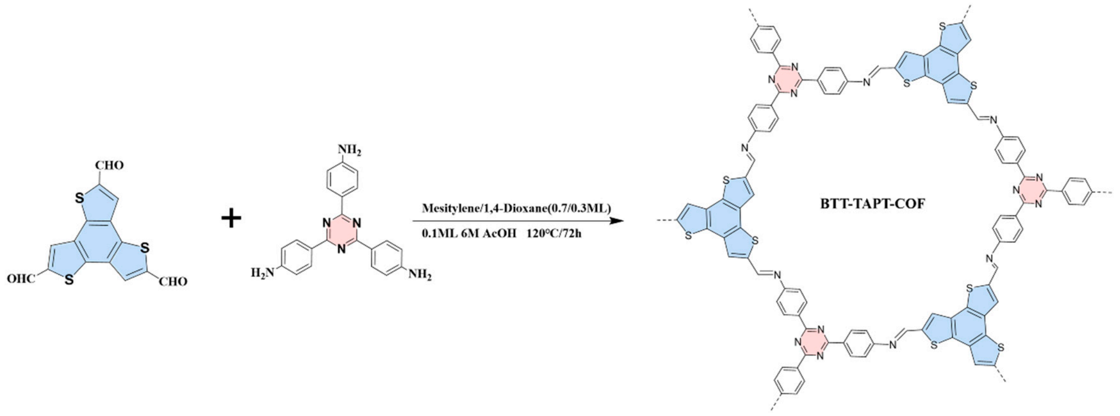

Figure S3. Synthesis of BTT-TAPA

8. Characterizations

8.1 FT-IR spectra

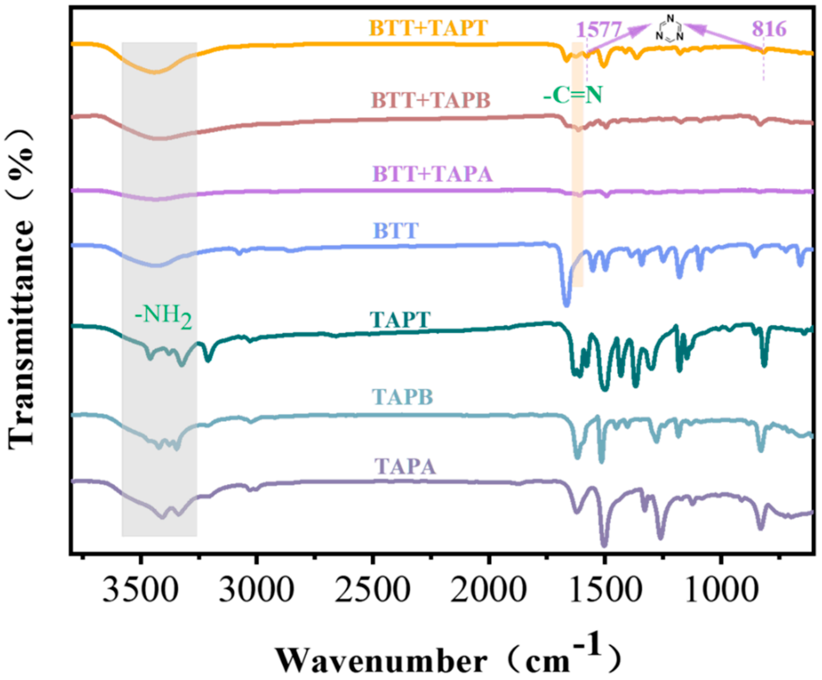

Figure S4. FT-IR spectra of BTT-TAPA, BTT-TAPB and BTT-TAPT

8.2 XPS spectra

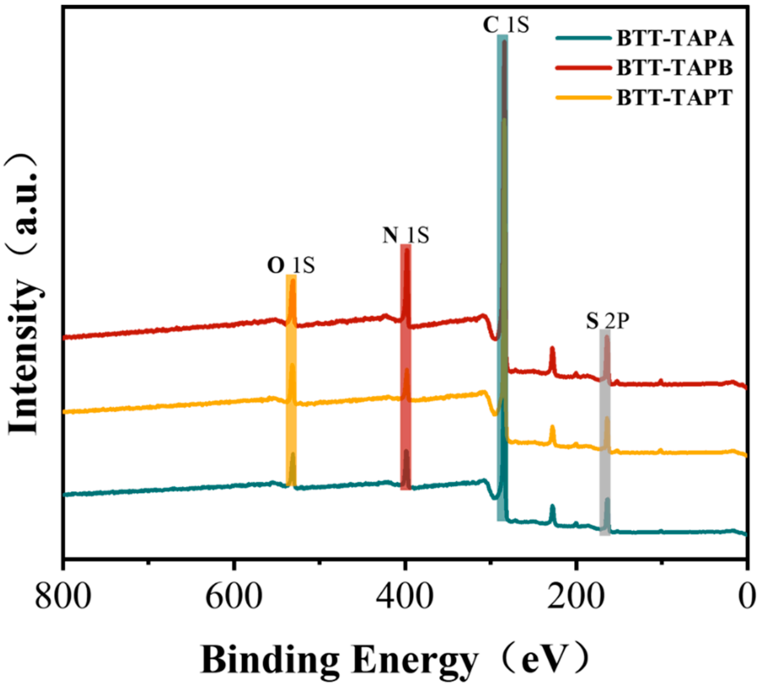

Figure S5. XPS spectra of BTT-TAPA, BTT-TAPB, and BTT-TAPT

8.3 Repetitive experiment of photodegradation

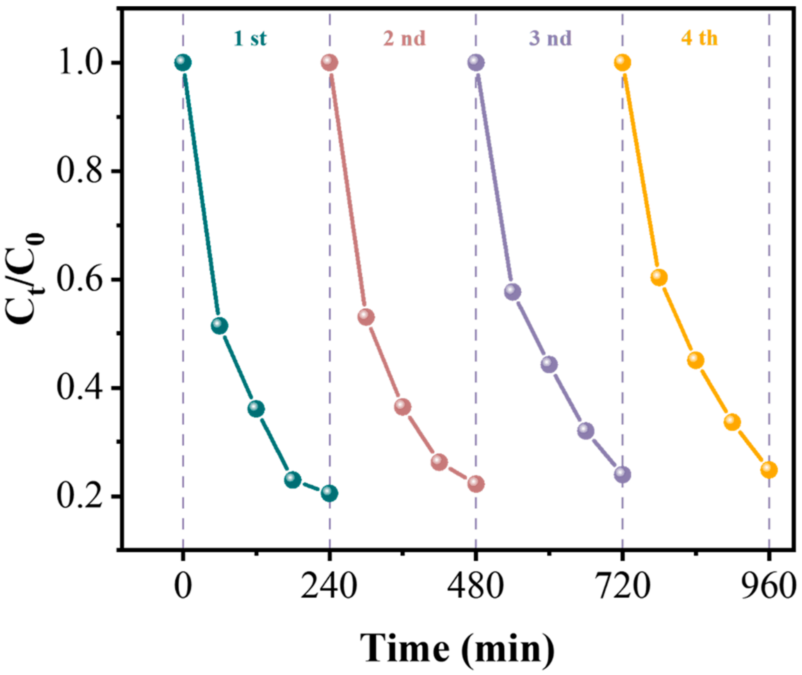

Figure S6. Repetitive photodegradation of BTT-TAPT on TC

8.4 EPR spectrum

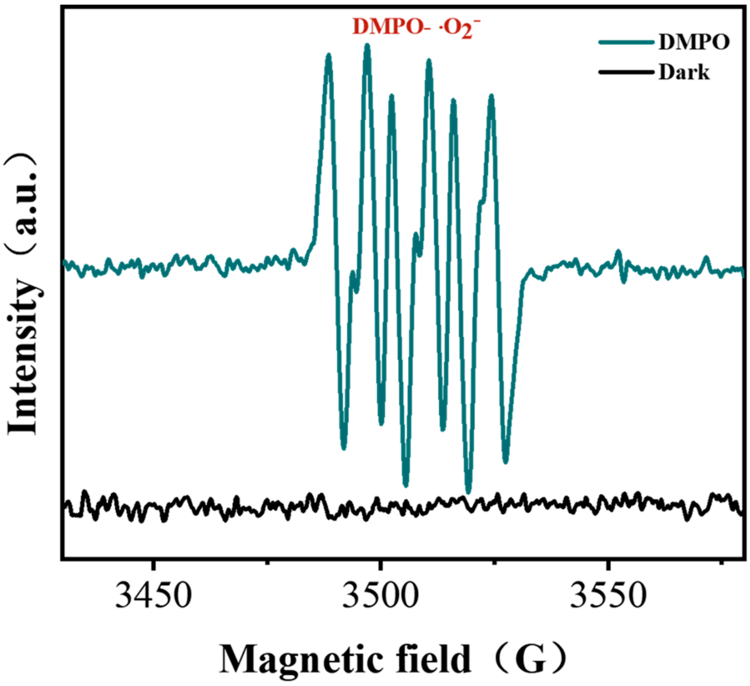

Figure S7. EPR spectra of BTT-TAPT.

8.5 High-resolution mass spectrometry

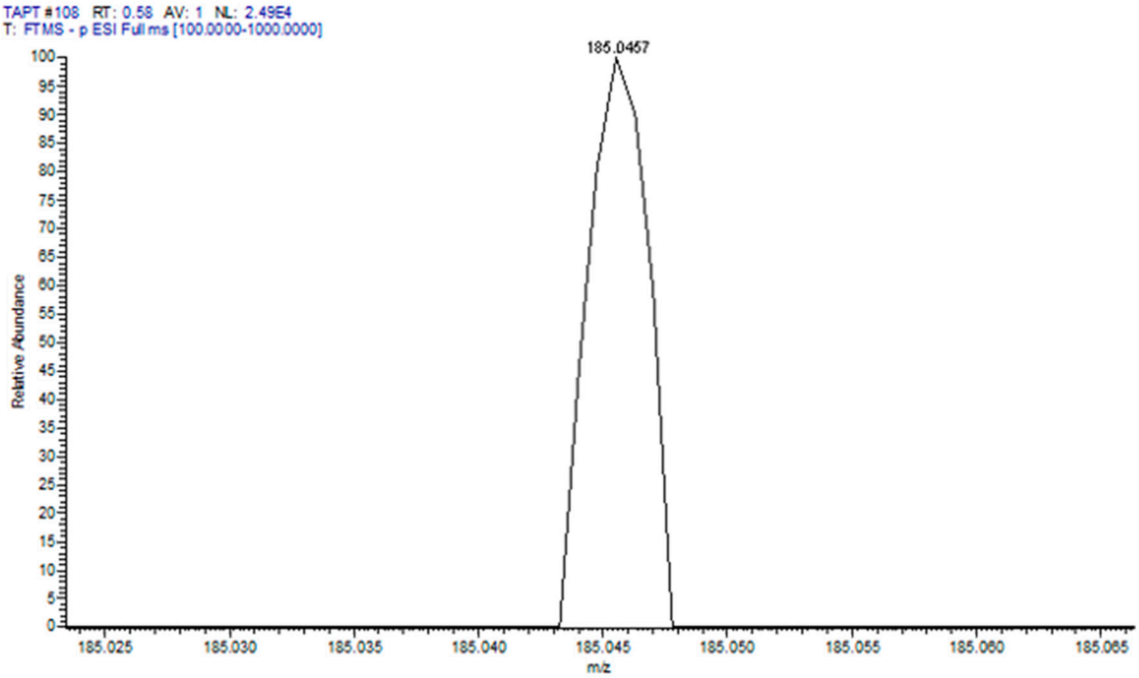

Figure S8. High-resolution mass spectrometry of DMPO with superoxide radicals.
